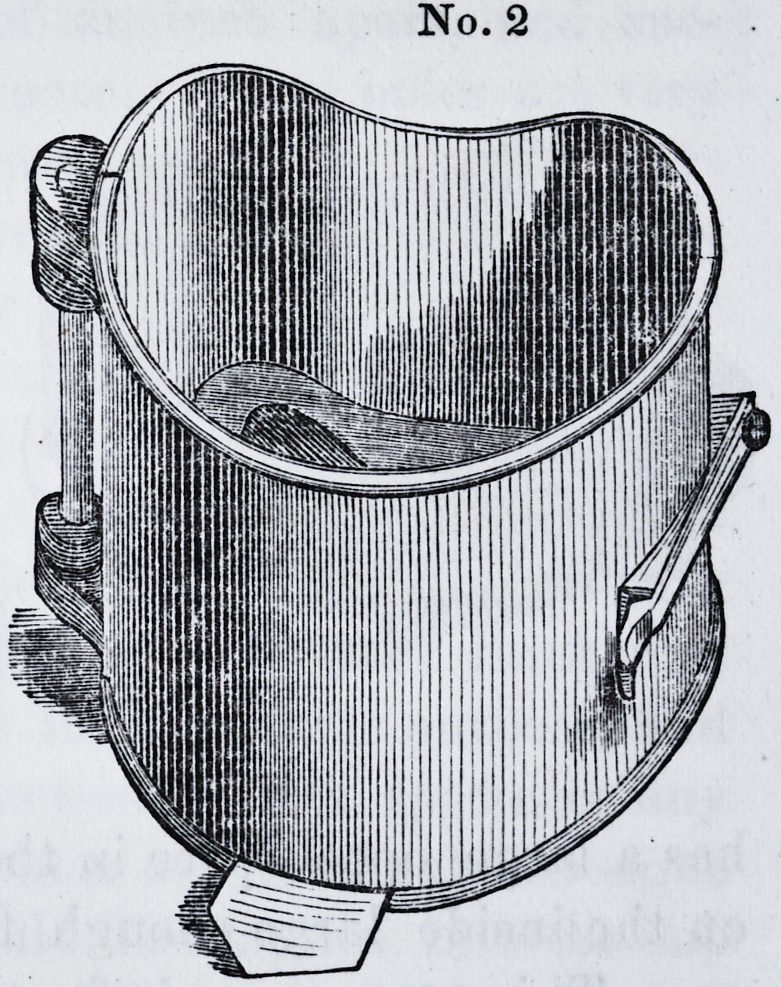# A New Method of Making Dies

**Published:** 1860-04

**Authors:** F. Y. Clark

**Affiliations:** Savannah, Ga.


### ARTICLE VIII.

*A New Method of Making Dies.* By F. Y. Clark, of Savannah, Ga.

To view comparatively the process for obtaining metallic dies, which we now offer the profession, we might speak at some length of the many objectionable features in connection with the various plans now in use; but, as we are addressing the experienced practitioner more than the student, we trust that these features are sufficiently familiar and understood to need no more than a mere allusion.

We believe there are very few, if any, who have not felt the want of a less tedious, more accurate, and simple process, while going through the disagreeable list of difficulties and manipulations, such as varnishing and oiling impressions, taking and trimming casts, varnishing again, and moulding in sand, using flasks and similar auxiliaries. If an irregular, prominent, or diverging alveolus is presented, a little skill and the yielding nature of the membrane will always enable us to secure a good impression; but, by our usual mode of procedure, a perfect metallic die of that impression is impossible. We may consume time, weary our heads and hands with flasks, pour the metal on our cast, or dip the cast in the metal, yet withal, as the principle is incorrect, the result must be the same.

We are confident that the most experienced portion of the profession will fully coincide with us in saying that what is wanted *is some simple method by which the metallic die can be taken directly from the impression.* Plaster comes nearer to what is wanted in this respect than any thing else in use , but were we to pour metal into a plaster impression, we know that the moisture would destroy the die: and to attempt to drive off this moisture by heat, would prove equally fatal to the impression. To accomplish our end, then, we found it necessary to forsake the beaten path pursued for years, and start anew, to seek some material, or combination of materials, by which we could secure a perfect impression, and which would resist the amount of heat necessary for obtaining a metallic die.

During the last three or four years our experiments to this end have been numerous. After testing from time to time, with more or less success, one material after another, and finally securing a combination that met our most sanguine expectations, we then found it necessary to construct a cup and flask, or set of flasks, by which this could be conveniently and successfully used for the end in view. In doing the latter, we have had much trouble, and spent much more time and money than many would suppose. We have gone from sheet iron, forced in shape by wooden forms, to copper struck up by iron dies, and then to various kinds of castings, until we arrived at that which we now offer, and which we believe, after being once fairly used, will never be thrown aside for any other method now in vogue.

With those prefatory remarks, we submit the following process, which we hope will be found as successful in the hands of others as it has been in our own:

To commence, it is necessary to have an impression-cup made from brass, German silver, copper, or any other metal that will stand the necessary amount of heat for obtaining a metallic die without change: we prefer one made from copper, because this metal is more malleable and easily cleaned after using, than any other that we have tried. It should differ in no other respect from the ones in general use, except in the perforation of holes all over its surface, about one-fourth of an inch apart, and one-eighth of an inch in circumference. These holes are very essential, for they not only prevent the material from leaving the cup in removing it from the mouth, but greatly facilitate the escape of moisture in drying or on receiving the metal for the die. With a cup as described, we use a batter composed of equal parts of clear, white spar and the best calcined plaster of paris. The manner of taking an impression with this batter is nearly the same as with plaster alone. It should be mixed somewhat thicker at first than plaster-batter used for the same purpose, and should be kept in constant motion with a spoon, or any thing that will answer, until there are unmistakable signs of its setting, and then it should be emptied into the cup and conveyed to the mouth as quickly as possible. As we said before, the plaster used for this purpose should be of the very best quality and freshly calcined, for it will not do to add salt to it to quicken its hardening, as is customary in the use of plaster alone. In very difficult cases, where the gums have receded, thereby exposing the necks of the teeth, it is best to have more spar than plaster in the batter used, for then the giving will be more apt to take place at the very point of difficulty. In such cases we know it is out of the question to get a perfect impression with anything; but we are confident this will be found more practicable than either wax or plaster, for it being harder than one and not as hard as the other, and somewhat brittle, is thereby more liable to give where it should—at the very point of trouble. We think a little experience is all that is necessary to convince any one that there are few cases, if any, where a better impression cannot be taken with this mixture than with either plaster or wax; for about the last two years we have used nothing else. The impression, when taken, and as represented in letter

A, should next be placed in flask No. 1, as represented in the following cut. This flask is cast from gray iron, and

No. 1 A

has a large open space in the bottom, leaving only a rim on the inside large enough for the impression-cup to rest on. This open space is for the escape of moisture coming from the holes in the cup, as before described. The space between the impression and the flask should now be seamed up with batter a little thicker than that used for the impression; it should be spread with a knife or spatula, so as to prevent it running down much between the cup and the rim of the flask. It is not best to use much batter for this purpose, just enough to hold the cup in place, and to give a smooth, continuous surface to the parts. When this is properly done it will present the appearance of the annexed representation, marked B. This flask and impression, thus prepared, is now ready for flask No. 2, which, when placed around the first, will present the following appearance. The whole should now be placed in an oven or on a stove, or any place where it will dry; this can be done either rapidly or gradually; when we are in a hurry we generally have it dry by the time the metal is melted, but it may be better to give it more time. It is not my wish to say anything here about metal; almost every dentist has some peculiar favorite of his own; of course it is immaterial what kind is used in this process. We generally construct our dies from zinc. Some time after the metal has been poured in the flask, a red hot iron should be

B No. 2

placed in its center, and held there until it begins to set, then the iron should be withdrawn and more metal poured in. This is done to command the shrinkage, making it take place where it will do no harm ; in other words, the metal should always be made to congeal last at the top of the flask : an iron, shaped like a tinner's soldering iron, is about the kind necessary for this purpose. When the die has become sufficiently cold, it should next be removed from the impression, etc., and flask No. 2 placed around it as before, then inverted, and the counter-cast taken.

In conclusion, we would remark, that all improvements should be considered valuable in proportion as they save time, money and labor. The time and labor necessary for obtaining a die by this process, will, on fair trial, be found about one-third of that consumed by the usual method, and the material, flasks, etc., will cost but a mere trifle—ten or twelve dollars. Here, then, as we economize time, money and labor, we think we may be allowed to assert *that we have an improvement.*

## Figures and Tables

**No. 1 f1:**
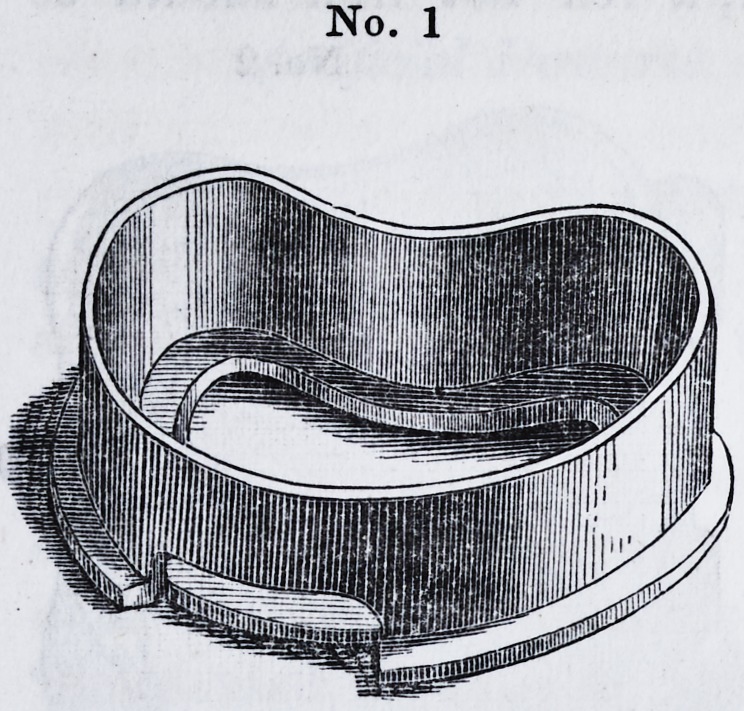


**Figure f2:**
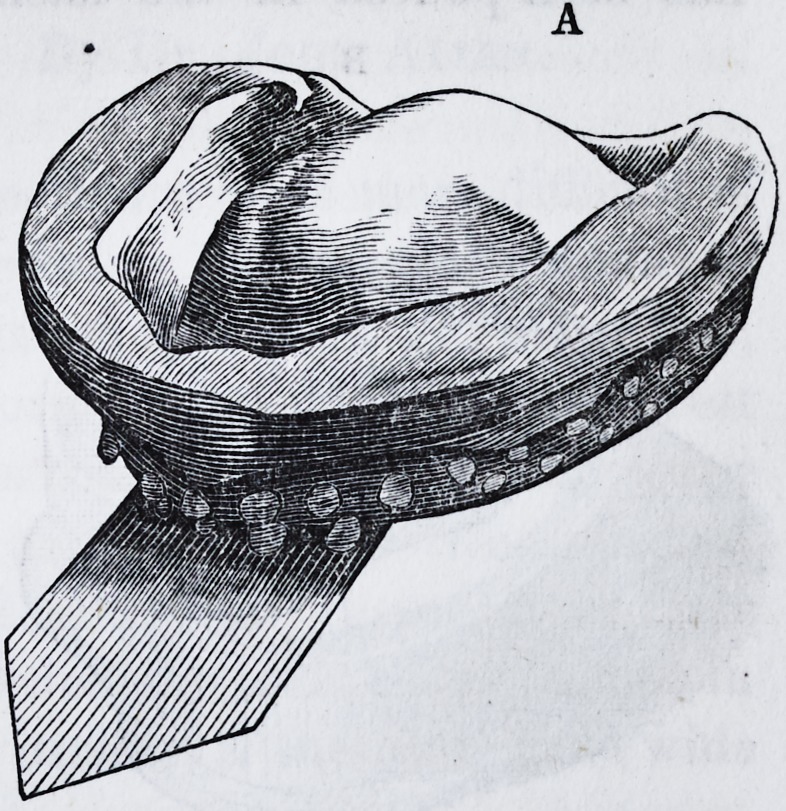


**Figure f3:**
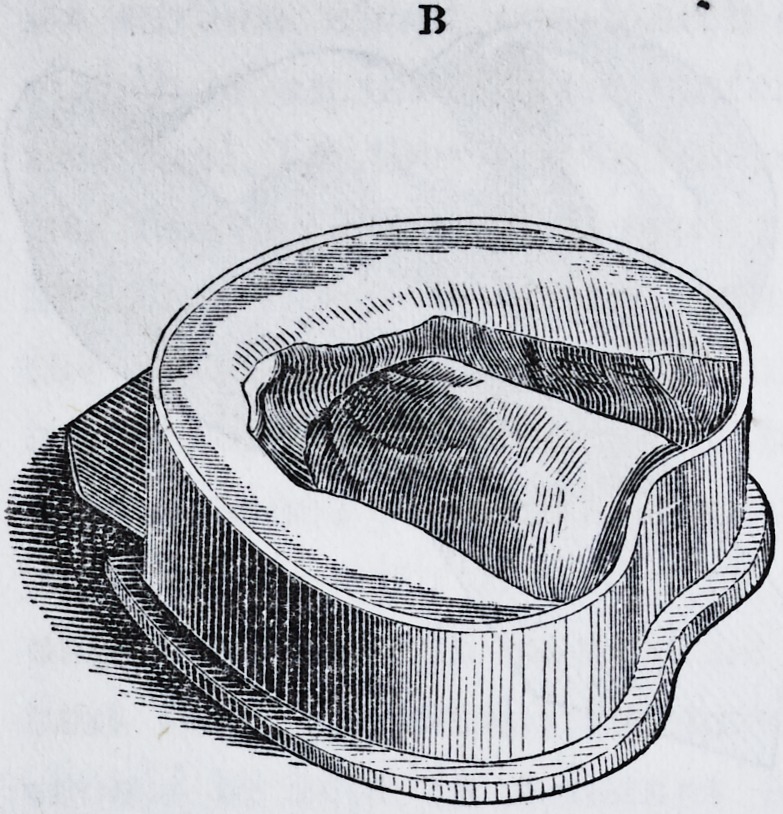


**No. 2 f4:**